# Regional femoral bone blood flow rates in laying and non-laying chickens estimated with fluorescent microspheres

**DOI:** 10.1242/jeb.242597

**Published:** 2021-08-20

**Authors:** Qiaohui Hu, Thomas J. Nelson, Roger S. Seymour

**Affiliations:** School of Biological Sciences, University of Adelaide, Adelaide, SA 5005, Australia

**Keywords:** Allometry, Bone blood flow, Microsphere infusion, Calcium mobilization, Metabolic rate, Nutrient foramen

## Abstract

The metabolic rate of vertebrate bone tissue is related to bone growth, repair and homeostasis, which are all dependent on life stage. Bone metabolic rate is difficult to measure directly, but absolute blood flow rate (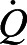) should reflect local tissue oxygen requirements. A recent ‘foramen technique’ has derived an index of blood flow rate (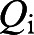) by measuring nutrient foramen sizes of long bones. 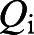 is assumed to be proportional to 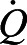; however, the assumption has never been tested. This study used fluorescent microsphere infusion to measure femoral bone 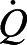 in anaesthetized non-laying hens, laying hens and roosters. Mean mass-specific cardiac output was 338±38 ml min^−1^ kg^−1^, and the two femora received 0.63±0.10% of this. Laying hens had higher wet bone mass-specific 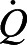 to femora (0.23±0.09 ml min^−1^ g^−1^) than the non-laying hens (0.12±0.06 ml min^−1^ g^−1^) and roosters (0.14±0.04 ml min^−1^ g^−1^), presumably associated with higher bone calcium mobilization during eggshell production. Estimated metabolic rate of femoral bone was 0.019 ml O_2_ min^−1^ g^−1^. Femoral 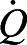 increased significantly with body mass, but was not correlated with nutrient foramen radius (*r*), probably because of a narrow range in foramen radius. Over all 18 chickens, femoral shaft 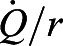 was 1.07±0.30 ml min^−1^ mm^−1^. Mean 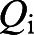 in chickens was significantly higher than predicted by an allometric relationship for adult cursorial bird species, possibly because the birds were still growing.

## Abstract (precis)

**Summary:** Femoral bone blood flow, measured using fluorescent microspheres, is approximately two times higher in laying hens than in non-laying hens and roosters. Blood flow values were related to foramen sizes.

## INTRODUCTION

Contrary to their commonly perceived status as inert, bones are highly vascularized to support processes that alter bone mass and structure such as modelling (growth) and remodelling (repair). In addition to cortical and trabecular bone that function for support and locomotion, birds develop medullary bone, particularly in the legs, to act as a labile calcium source for eggshell formation around the onset of sexual maturity (Whitehead, 2004). Commercial layer chickens require a lot of calcium for intense egg production, and about 10% of their total body calcium is cycled into the shell of every egg produced (Bar, 2009). Blood carries calcium as well as oxygen, hormones and nutrients to bones to satisfy varied metabolic demands. Studying regional blood flow rates improves the understanding of physiological processes that occur in different organs, because the oxygen demand of an organ generally determines the blood supply to it (Wolff, 2008). Studying bone blood flow in chicken femoral bones can thus provide insight into chicken femur metabolic demand associated with physiological processes.

Measuring regional perfusion is challenging, but techniques such as Doppler ultrasound and microsphere infusion have been developed over the last century. Microsphere infusion has been particularly useful to quantify regional blood flow and blood flow distribution. This technique requires injection of microspheres into the left ventricle or atrium of an animal, and it relies on the principle that the microspheres are distributed evenly within the bloodstream after injection and lodged in the microcirculation. The number of microspheres that are trapped in tissue capillaries or small arteries is proportional to the regional perfusion rate (Anetzberger and Birkenmaier, 2016). Absolute regional blood flow rates can be measured, following the invention of the arterial reference sampling technique, which uses a pump as an artificial organ to withdraw arterial blood at a constant, known rate from the same experimental animal during microsphere injection (Kaihara et al., 1968; Makowski et al., 1968; Neutze et al., 1968). Fluorescent microspheres have been specifically used to quantify blood flow in bones (Anetzberger et al., 2004b; Aref et al., 2017; Serrat, 2009).

Another method to estimate regional blood flow rate is to measure the sizes of foramina that contain blood vessels in bones. This ‘foramen technique’ relies on a theory that foramen size is proportional to the size of the occupying vessels. This technique has been developed to evaluate the blood supply to femora through nutrient foramina (Allan et al., 2014; Hu et al., 2018; Schwartz et al., 2018; Seymour et al., 2012) and to brains through carotid foramina (Boyer and Harrington, 2018, 2019; Seymour et al., 2015, 2016, 2019). Blood flow rates estimated from human, rat and mouse carotid foramina match direct blood flow measurements (Seymour et al., 2015), suggesting this technique can provide accurate regional perfusion values in some cases by simply measuring the size of foramina. However, no nutrient foramen studies have ever related femoral nutrient foramen size to absolute blood flow of femoral bone, because the nutrient artery does not completely fill the nutrient foramen. Therefore, the foramen technique results in a blood flow index (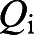) only, but it is assumed to be proportional to absolute blood flow rate (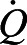).

The present study had two objectives. The first was to correlate 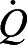 through femoral nutrient foramina and 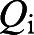 estimated from foramen size, by comparing the microsphere infusion technique and foramen technique. The second was to evaluate femoral bone perfusion in chickens with both techniques. Three chicken groups (i.e. non-laying hens, laying hens and roosters) with similar ages were chosen as experimental animals as only a few studies have looked into femoral bone blood flow in birds (Boelkins et al., 1973). Laying hens and non-laying hens were compared to test the hypothesis that layers exhibit higher rates of femoral bone blood flow because of the role of medullary bone in eggshell formation (Whitehead, 2004).

## MATERIALS AND METHODS

### Animal preparation

Crossbreed ISA brown hens and roosters aged 4–8 months were used in this study. Animals were obtained under Animal Ethics Committee approval (S-2017-058). Chickens were divided into three groups: non-laying hens, laying hens and roosters. Each group consisted of six individuals. Chickens were kept in a constant temperature room (25°C) with a 16 h day:8 h night cycle before operations. All chickens had free access to water and calcium-rich food. Hens that were sexually immature and had not developed any eggs in their reproductive organs were designated as non-laying hens, and usually they were not older than 5 months. Hens that had begun to lay eggs regularly were selected as laying hens, and their ages ranged from about 6 to 8 months. Ages of roosters ranged from 4 to 7 months.

### Microsphere standard curve

Polystyrene, green fluorescent (excitation wavelength: 450 nm; emission wavelength: 480 nm), 15 µm microspheres (FluoSpheres, Thermo Fisher Scientific) were used for determination of cardiac output and blood flow rate of femoral bone. Before using each microsphere vial for injections, the relationship between fluorescence intensity and microsphere number/concentration was determined by dissolving known amounts of microsphere suspensions in cellosolve acetate (2-ethoxyethyl acetate, 98%, Sigma, cat. no. 109967-1L), and by analysing fluorescence intensity of a series of cellosolve acetate solutions with different concentrations of dissolved microspheres using a Cary Eclipse Fluorescence Spectrophotometer (Varian Australia Pty Ltd). An excitation wavelength of 450 nm and a slit width of 10 nm were chosen.

### Procedures

A 2 ml plastic syringe with a 25 gauge needle was prepared for microsphere injection. This syringe and the needle were weighed separately to 0.0001 g. A 2 ml glass syringe was filled with 1 ml heparinized saline (125 i.u. ml^−1^) and placed on a syringe pump (Harvard Universal Infusion Pump, Harvard Apparatus, Holliston, MA, USA), modified to withdraw reference blood during microsphere injection.

Before each operation, chickens were weighed to 1 g. They were anaesthetized with a combination of ketamine (40 mg kg^−1^) and xylazine (4 mg kg^−1^). Under anaesthesia, they were placed on their right side and were stabilized on a dissection table using a wooden frame with Velcro. Chickens were rested on a thick, dry towel to keep them warm, and the room temperature was 25°C. Feathers at the left humerus region were plucked, and the skin next to the wing brachial vein was removed. A scalpel was used to separate the biceps and triceps next to the brachial vein to expose the brachial artery underneath the muscles. The brachial artery was then isolated and blocked at the proximal region with a temporary ligature. The brachial artery was cannulated and sutured distal to the ligature and toward the heart using heparinized clear vinyl tubing (inner diameter: 0.5 mm; outer diameter: 0.8 mm) with a heparinized 25 gauge needle connected to the end.

Immediately after artery cannulation, a microsphere vial was vortexed for 10 s and sonicated for 4 min in an ultrasonic cleaner (Bransonic B-221, Branson Cleaning Equipment Company, Shelton, CT, USA). During the sonication, the chicken pericardium was exposed by cutting into the left pectoralis major muscle and opening the gap between the first second and third ribs. A heparinized 20 gauge Venocan pencil style i.v. catheter (cat. no. 121931, Kruuse, Langeskov, Denmark) was inserted into the left ventricle and connected to a pressure transducer (P23Dc, Statham Instruments, Hato Rey, Puerto Rico) and amplifiers (Model 79D EEG, Grass Instruments, Quincy, MA, USA). The output of the equipment was recorded to a computer with an analog–digital converter and software (DI-145, WinDaq version 3.98, DATAQ Instruments, Akron, OH, USA). As the catheter needle tip reached the left ventricle, a typical left ventricular tracing wave could be observed. Flow in the brachial artery was then restored at the proximal region by removing the temporary ligature. More heparinized saline was injected into the brachial artery if blood did not flow out to the cannulated vinyl tubing because of blockage. The other end of the tubing with the 25 gauge needle was then connected to the 2 ml glass syringe on the syringe pump. Blood was continuously withdrawn from the brachial artery from 30 s before microsphere injection until 2 min after it. The withdrawal rate was set at either 0.28 or 0.46 ml min^−1^, depending on the size of the chicken. A 1.5 ml sample of sonicated microsphere suspension (∼1.5×10^6^ microspheres) was withdrawn into the preweighed 2 ml plastic syringe. The syringe was weighed again to 0.0001 g, and the needle was removed for later weighing. The catheter needle was removed from the catheter inside the left ventricle, and the 1.5 ml microsphere suspension was slowly injected into the left ventricle over 15 s. Chickens were killed by injecting excessive anaesthetic into the left ventricle through the catheter, 2 min after the microsphere injection. To account for uninjected microspheres, the 2 ml plastic syringe was rinsed out with 2% Tween 80 into a 40 ml glass vial, and the needle with uninjected microspheres was weighed again and placed into another 4 ml glass vial for later fluorescence intensity analysis.

### Sample processing

Some of our processes referred to a recent protocol, which describes in detail a method to measure relative bone blood supply in mice with fluorescent microspheres (Serrat, 2009).

Reference withdrawal blood in the glass syringe was poured into a 100 ml glass bottle. The glass syringe was rinsed 3 times using ∼20 ml 2% Tween 80, and all rinse liquid was also poured into the 100 ml glass bottle. A further 2 ml of heparinized saline was added into the bottle to prevent the blood from clotting.

The spleen and kidneys of five laying hens were harvested, weighed and kept in phosphate buffered saline (PBS) in the dark before tissue digestion and microsphere analysis to determine organ blood flow for comparison with the literature. Femora were harvested, and nutrient foramen microphotographs were taken using a microscope set up. Fiji (open source, www.fiji.sc) was used to measure the foramen areas to calculate foramen radii. Methods that measure foramen size microphotographically are described in detail elsewhere (Hu et al., 2020). Femur lengths were measured to 1 mm. Nutrient arteries support the whole femur shaft region while the metaphyses and epiphyses receive perfusion from other arteries (Trueta, 1963). Therefore, we expected that nutrient foramen sizes would be more related to flow to the shaft region rather than to the whole femur. Femora were therefore sectioned into three parts, as previously illustrated (Aref et al., 2017; Colleran et al., 2000): the proximal end (25%), shaft (42%) and distal end (33%), measured in relation to total femur length. Bone marrow was retained in all sections. Each bone section was weighed to 0.001 g and then placed into Cal-Ex decalcifying solution in the dark for 4–5 days. After decalcification, bone samples were rinsed 3 times with PBS and placed into 100 ml glass bottles. Freshly prepared 100 ml quantities of 2 mol l^−1^ ethanolic KOH with 2% Tween 80 were used to digest all blood, soft tissue and decalcified bone samples. The glass bottles were placed on a swirling shaker (No. 436, Penetron Mark III, Sunkay Laboratories, Inc., Tokyo, Japan) in the dark for 2–3 days to allow sample digestion to take place; the digestion process was completed when there were no large particles remaining in the bottles.

Digested tissues and blood were filtered using a glass vacuum filtration apparatus with glass microfiber 1.2 µm filter paper (Grade 333, 47 mm diameter; Filtech, Wollongong, NSW, Australia). During filtration, 2% Tween 80 was used to rinse the sample bottles 3 times and at least 100 ml potassium phosphate buffer rinse solution was used for the final rinse of the filtration unit and to adjust pH. After filtration, filter papers were moved and pushed into 70 ml, flat bottom vials (diameter: 44 mm; height: 57 mm) using forceps and polyethylene plungers. The plungers remained inside the vials and the vials were placed in the dark before fluorescence intensity analysis. On the day of analysis, 12 ml cellosolve acetate was added into each vial to dissolve the microspheres and release fluorescent dye. Vials were vortexed well and placed in the dark for 2–4 h before analysis. Three replicates of 3 ml solutions from each sample vial were pipetted into glass cuvettes for analysis. If the fluorescence intensity was higher than the upper record limit of the spectrophotometer, the samples were quantitatively diluted with cellosolve acetate in the cuvette to make up 3 ml solutions. Uninjected microspheres in the injection syringe and needle were also quantified.

### Microsphere analysis

A pilot study was conducted to investigate whether our experimental setups would cause severe microsphere loss during processing. Known numbers of microspheres were placed in different glass vials with either Cal-Ex or 2 mol l^−1^ ethanolic KOH solutions for 2–4 days, and the microspheres were filtered and dissolved in cellosolve acetate solutions. The number of recovered microspheres was quantified. We found that almost all microspheres (>95%) were successfully recovered. Fluorescence intensity detected in samples was converted to number of microspheres. The number of injected microspheres for each chicken was calculated and calibrated based on microsphere suspension density (1.005 g ml^−1^), the mass difference between the syringe and needle with and without microspheres, and the amount of uninjected microspheres in the injection syringe and needle. Cardiac output from the left ventricle (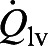, ml min^−1^) of each chicken was calculated as: 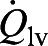
=(*V̇*_with_×*N*_inj_)/*N*_blood_, where *V̇*_with_ is pump withdrawal rate (ml min^−1^), *N*_inj_ is the number of injected microspheres and *N*_blood_ is the number of microspheres in the reference blood sample. Absolute blood flow rate (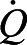, ml min^−1^) to different tissues was calculated by the equation: 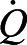
=(*V̇*_with_×*N*_tis_)/*N*_blood_, where *N*_tis_ is the number of microspheres recovered from the target tissue. Femoral bone blood flow rates were averaged from both left and right femora for each individual chicken.

### Statistical analysis

All error statistics refer to 95% confidence intervals (CI) calculated in GraphPad Prism 6.0 (GraphPad Software, La Jolla, CA, USA).

Mass-specific cardiac output (ml min^−1^ kg^−1^) and tissue blood flow rate (ml min^−1^ g^−1^) were calculated by dividing the absolute blood flow rate by body mass (kg) and individual wet tissue mass (g), respectively. Foramen area (mm^2^) and radius (mm) of each chicken were averaged from both femora. Body mass, mass-specific cardiac output, blood flow rate, foramen area and foramen radius among three chicken groups were compared using ANOVA. If ANOVA showed a significant difference among groups, Tukey's multiple comparisons test was used for comparing means between two groups. The 95% CI of the differences between the two groups were also calculated. Mass-specific blood flow rate is commonly used in literature, so we used it here. We recognize that most biological factors scale with body mass in non-linear ways, thus mass-specific values may be still dependent on body mass. True mass-independent data (raw values divided by body mass raised to the exponent of the allometric equation describing the relationship between the values and body mass) are ideally used, and some are presented in Supplementary Materials and Methods, Figs S1 and S2, and Table S1. However, in this study, there was little difference between mass-specific and mass-independent data.

Femoral bone blood flow index (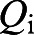) was calculated using an equation derived from Poiseuille's law: 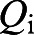
=*r*^4^/*L*, where *r* (mm) is the foramen radius and *L* (mm) is an arbitrary length, measured as femur length (Allan et al., 2014; Hu et al., 2018; Seymour et al., 2012). It was assumed that the arbitrary units of 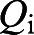 (mm^3^) are proportional to absolute blood flow rate. To compare 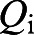 between chickens and other cursorial birds interspecifically, nutrient foramen sizes of adult chickens would be required. However, our chickens were not mature enough to be considered as fully grown adults, especially non-laying hens. Therefore, the analysis is based on body mass, not age. To present chicken 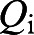 more precisely, only laying hens and roosters were selected to estimate 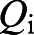 as they were generally older than the non-laying hens and were close to being adults. Nutrient arteries mainly supply the femur shaft. To test whether foramen size is associated with blood flow rate, Pearson's correlation coefficient (Pearson's *r*) was calculated to measure the strength of a linear correlation between foramen radius and femur shaft blood flow rate in all 18 chickens.

## RESULTS

The body mass of the 18 chickens ranged from 1.1 to 2.7 kg, and the mean body mass was 1.67±0.22 kg. Body mass was significantly different among the three groups (*F*_2,15_=8.4, *P*=0.004), being significantly lower in non-laying hens (1.25±0.11 kg) than in laying hens (1.78±0.20 kg; *P*=0.03, 95% CI of the difference −0.54±0.48 kg) and roosters (2.0±0.54 kg; *P*=0.004, 95% CI of the difference −0.73±0.48 kg). The mean and 95% CI of body mass-specific cardiac output of the three chicken groups was 338±38 ml min^−1^ kg^−1^; ANOVA showed no significant differences among the three groups (*F*_2,15_=0.10, *P*=0.90). Average spleen and kidney masses of five laying hens were 1.8±0.4 and 10.7±2.9 g, respectively. Data collected incidentally showed that the spleen of laying hens received 1.6±1.0% of cardiac output and required 5.6±5.3 ml min^−1^ g^−1^ of blood flow. The kidneys received 4.2±1.0% of cardiac output and required 2.4±1.4 ml min^−1^ g^−1^ of blood flow.

**Fig. 1.**
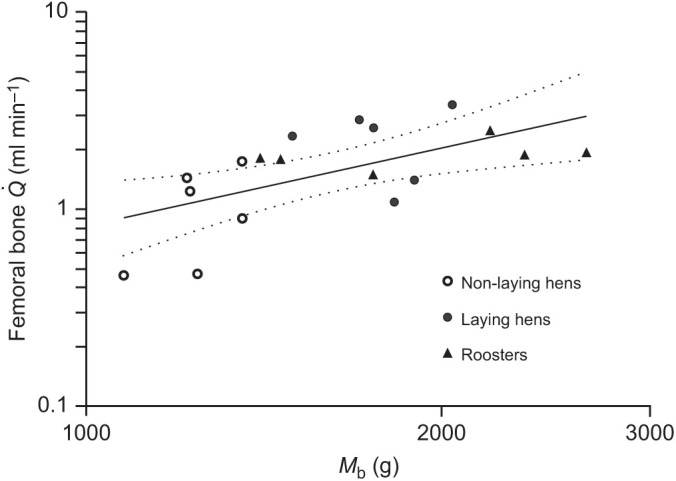
**Relationship between femoral bone blood flow rate (**
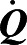
**) and body mass (*M*_b_) for non-laying hens, laying hens and roosters**. The equation set to all groups was 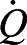
=9.1×10^–5^*M*_b_^1.32±0.93^ (*R*^2^=0.36; *P*=0.008). Dashed lines represent the 95% confidence intervals for the regression mean. Data are plotted on logarithmic scales.

**Fig. 2.**
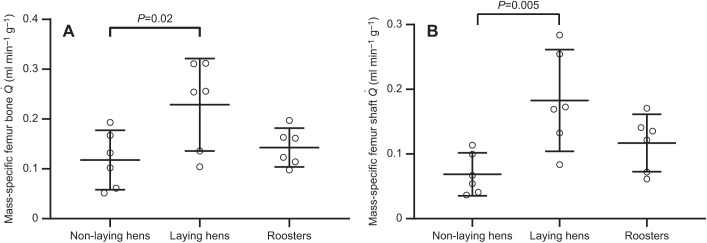
**Mass-specific femur blood flow rate among non-laying hens, laying hens and roosters.**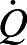 is shown for the entire femur (A) and the femur shaft (B). Mass is wet bone mass. Error bars represent the 95% confidence interval (CI) of the means of 6 replicates.

Blood flow rate to the femoral bone was not significantly related to body mass when all three groups of chickens were considered, as the 95% CI of the exponent (1.32±0.93) included 1.0 (Fig. 1). The slope was significantly different from 0 (*R*^2^=0.36, *P*=0.008). Comparison of the scaling relationships among the three chicken groups by ANCOVA indicated that there were no significant differences in the scaling exponents (*F*_2,12_=1.2, *P*=0.33) and scaling factors (*F*_2,14_=1.8, *P*=0.20). However, mass-specific values showed that laying hens had significantly higher blood flow rates to the whole femoral bone and to the shaft than the non-laying hens (whole femur: *P*=0.02, 95% CI of the difference −0.11±0.10 ml min^−1^ g^−1^; femur shaft: *P*=0.005, 95% CI of the difference −0.11±0.08 ml min^−1^ g^−1^) (Fig. 2A,B). Total and regional femoral bone blood flow of the non-laying hens and roosters were not significantly different from each other (Table 1). Mass-independent femoral bone blood flow rate and shaft blood flow rate compared among the three chicken groups showed results similar to the mass-specific value comparisons (Fig. S1 and Table S1).

**Table 1.**
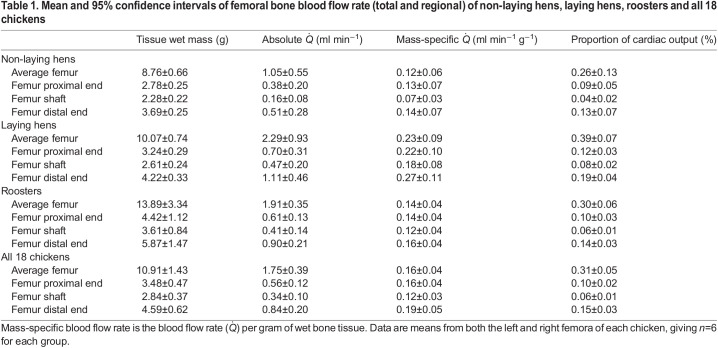
Mean and 95% confidence intervals of femoral bone blood flow rate (total and regional) of non-laying hens, laying hens, roosters and all 18 chickens

Averaged across all chickens, the two femora received a combined total of 0.63±0.10% of cardiac output. Within the individual femora of six non-laying chickens, the proximal end received 35.7±1.6%, the shaft 15.7±2.7% and the distal end 48.6±3.2% of the total femoral bone blood flow. Laying hens received 30.5±4.8%, 20.9±3.4% and 48.6%±5.5%, respectively, and roosters received 31.7±4.6%, 21.1%±5.8% and 47.1±6.4%, respectively.

**Fig. 3.**
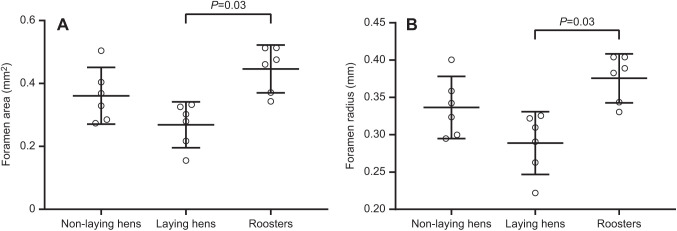
**Nutrient foramen area and radius of non-laying hens, laying hens and roosters.** Foramen area (A) and radius (B). Error bars represent the 95% CI of the means of 6 replicates.

To estimate chicken femoral bone blood flow index (*Q*_i_), foramen sizes were averaged from both laying hens and roosters. Mean foramen radius was 0.33 mm and foramen area was 0.36 mm^2^ in the 12 laying hens and roosters. Average 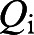 of these chickens was 1.50×10^−4^ mm^3^. Mean foramen area of non-laying hens, laying hens and roosters was 0.36±0.09, 0.27±0.07 and 0.45±0.08 mm^2^, respectively (Fig. 3A). Mean foramen radius of all three groups was 0.34±0.04, 0.29±0.04 and 0.38±0.03 mm, respectively (Fig. 3B). Laying hens had significantly smaller nutrient foramen sizes than the roosters (area: *P*=0.003, 95% CI of the difference −0.18±0.11 mm^2^; radius: *P*=0.003, 95% CI of the difference −0.09±0.06 mm), but foramen sizes were not significantly different between non-laying hens and laying hens (area: *P*=0.13, 95% CI of the difference 0.09±0.11 mm^2^; radius: *P*=0.10, 95% CI of the difference −0.05±0.06 mm) or between non-laying hens and roosters (area: *P*=0.16, 95% CI of the difference being −0.09±0.11 mm^2^; radius: *P*=0.20, 95% CI of the difference being −0.04±0.06 mm).

**Fig. 4.**
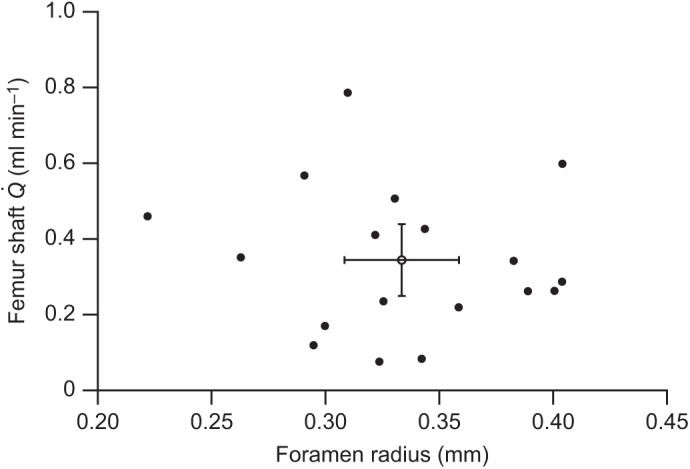
**Individual values of foramen radius and femur shaft blood flow rate in chickens from all groups.** Filled circles represent foramen radius and femur shaft blood flow rate of individual chickens (*n*=18; Pearson's *r*=−0.11, *P*=0.68); the open circle represents the mean and 95% CI.

Nutrient foramen radius and femur shaft blood flow rate showed no correlation in our study because Pearson’s correlation coefficient *r*=−0.11 and *P*=0.68 (Fig. 4). In all 18 chickens, mean blood flow rate to the shaft bone was 0.34±0.10 ml min^−1^ and mean nutrient foramen radius was 0.33±0.02 mm, giving a mean ratio of 1.07±0.33 ml min^−1^ mm^−1^.

## DISCUSSION

### Chicken cardiac output

The mass-specific cardiac output of our roosters (337.9±39.7 ml min^−1^ kg^−1^) was significantly higher than the value (150.4±28 ml min^−1^ kg^−1^) measured using radioactive microspheres in adult roosters under local anaesthesia (Merrill et al., 1981). The cardiac output of our laying hens (327.2±104.7 ml min^−1^ kg^−1^) was also significantly higher than the values (177±11 and 218 ml min^−1^ kg^−1^, respectively) reported by Boelkins et al. (1973) and Sapirstein and Hartman (1959) using an indicator dilution technique in adult laying hens. However, Boelkins et al. (1973) used two different dyes to measure mass-specific cardiac output, and the value they obtained using Evans Blue dye (277±16 ml min^−1^ kg^−1^) is not markedly different from our value. Therefore, methodological differences may cause a wide range of cardiac output values. Interspecifically, the scaling of cardiac output on the body mass of avian taxa is CO=290.7*M*_b_^0.69^, where CO is cardiac output (ml min^−1^) and *M*_b_ is body mass (kg) (Grubb, 1983). Grubb's cardiac output values were collected by measuring the arteriovenous oxygen content difference and oxygen consumption rate (Fick principle) under local anaesthesia. According to the equation, mass-specific cardiac output of chicken is estimated to be 241 ml min^−1^ kg^−1^ using our average chicken body mass (1.82 kg). This estimated mass-specific cardiac output value is significantly lower than our estimated value, but significantly higher than all the literature values of laying hens described above.

It is not clear why mass-specific cardiac output was somewhat high in our study, but it may be related to the fact that previous studies involved older chickens in which cardiac output would be expected to be lower. In humans, mass-specific cardiac output tends to decrease as body mass increases (de Simone et al., 1997). Body mass-specific cardiac output of broilers also decreased with ageing from 4 to 6 weeks, although this was not statistically significant (Wideman, 1999). The decline is mainly associated with decreasing mass-specific metabolic rate with growth, but it also may be influenced by body composition. Additionally, anaesthetics can affect animal heart rate and cardiac output, although the impact on birds is not fully understood. Ketamine alone can maintain or increase heart rate in birds while xylazine alone can reduce heart rate and respiration rate and may cause hypoxaemia, hypercarbia and death in birds (Abou-Madi, 2001; Varner et al., 2004). The ketamine/xylazine combination has been widely used to anaesthetize animals including birds, as xylazine relaxes muscles well, along with ketamine (Abou-Madi, 2001). However, the ketamine/xylazine combination still has side effects. For example, lower doses (ketamine: 15 mg kg^−1^; xylazine: 0.15 mg kg^−1^) can reduce the heart rate of the great horned owl (Raffe et al., 1993). Lowering body temperature may also affect cardiac output. For instance, spinal cord cooling and ambient cooling can increase the heart rate and cardiac output of pigeons by raising compensatory thermogenesis (Barnas et al., 1984). However, all of our surgical procedures were performed at room temperature, which is within the thermal neutral zone of chickens (Van Kampen et al., 1979). The duration from chest opening to microsphere injection was controlled to be as short as possible; however, exposing organs to a lower ambient temperature may still have influenced cardiac output.

### Absolute rate of blood flow in the chicken femoral bone

The percentage of blood flow to three femoral regions in chickens was similar to that in rats (Aref et al., 2017), with the two ends receiving more blood flow than the shaft. Wet bone mass-specific blood flow in the femoral bone of laying hens was 0.23±0.09 ml min^−1^ g^−1^, which is much lower than the 0.77±0.09 ml min^−1^ g^−1^ measured using radioactive microspheres in laying hens (Boelkins et al., 1973). Despite this difference, the spleen and kidneys of our laying hens received a wet tissue mass-specific blood flow of 5.6±5.3 and 2.4±1.4 ml min^−1^ g^−1^, respectively, which are not significantly different from the 4.81±0.95 and 2.48±0.26 ml min^−1^ g^−1^ reported by Boelkins et al. (1973). Wet bone mass-specific blood flow rate in the femoral bone of non-laying hens (0.12±0.06 ml min^−1^ g^−1^) and roosters (0.14±0.04 ml min^−1^ g^−1^) was not significantly different from the rate (0.13–0.15 ml min^−1^ g^−1^) measured in rabbit femora using both radioactive and fluorescent microspheres (Anetzberger et al., 2004a). Wet bone mass-specific flow rate in the proximal end, shaft and distal end of rabbit femora was approximately 0.16–0.17, 0.15 and 0.11–0.12 ml min^−1^ g^−1^, respectively (Anetzberger et al., 2004a), and these values were not significantly different from most of our regional femoral bone blood flow values in chickens, the shaft in non-laying hens and the distal end in roosters being the only exceptions (Table 1).

Blood flow rate can be used to roughly estimate metabolic rate of the supplied tissues. The haemoglobin content of chicken blood is about 0.18 g ml^−1^ (Elagib and Ahmed, 2011). Assuming each gram of haemoglobin carries 1.34 ml of oxygen (Hüfner's constant), and assuming half of blood oxygen is consumed by bone tissue, every millilitre of blood then supplies (1.34×0.18)/2=0.12 ml of oxygen to the bone tissue. Mean absolute blood flow rate of femoral bone is 1.75 ml min^−1^ and femur mass is 10.9 g. Therefore, the femur metabolic rate is estimated to be (0.12×1.75)/10.9=0.019 ml O_2_ min^−1^ g^−1^. This is about twice the metabolic rate (0.0095 ml O_2_ min^−1^ g^−1^) of adult guinea pig calvarial bone (Schirrmacher et al., 1997), but this is expected given that the chickens were measured *in vivo* at normal body temperature and the guinea pig bone was measured *in vitro* at room temperature. We are unaware of any published estimates of avian bone metabolic rate or oxygen extraction ratio.

### Bone blood flow and egg production

Laying hens had a significantly higher wet bone mass-specific femoral bone blood flow rate and femur shaft blood flow rate than the non-laying hens. Femoral bone blood flow of roosters was not significantly different from that of non-laying hens and laying hens, suggesting sex alone does not affect femoral bone blood flow in chickens around the onset of sexual maturity. However, egg production in laying hens may affect bone perfusion. The mean mass-specific blood flow rate of the femoral bone was approximately 1.9 times higher, and femur shaft blood flow rate was 2.7 times higher, in laying hens than in non-laying hens (Fig. 2). The major cause of this blood flow difference may relate to calcium homeostasis. Laying hens may require extra blood flow to transport oxygen, which is vital for medullary bone metabolism associated with calcium deposition and mobilization. As hens reach sexual maturity, osteoblasts in chicken leg bones start to form medullary bone, which is a special bone type that exists only in birds and crocodylians, and it acts as a labile calcium reserve for eggshell formation (Whitehead, 2004). During egg production, calcium can be removed and regained rapidly in medullary bone. To maintain calcium balance, laying hens need to consume a great amount of calcium in their diet. During the daytime when chickens are active, calcium from their diet is absorbed from the intestines and used directly for eggshell formation or stored in bone for later use. Almost no calcium is left in the intestines 6–10 h after feeding (Bar, 2009). Eggshell formation of laying hens is high during the night, when calcium is obtained from bone. The calcium loss in bones can then be regained the next day when laying hens absorb calcium from their food source. Laying hens need 2.2 g of calcium, which represents about 10% of total body calcium, for daily egg production (Bar, 2009; Bouvarel et al., 2011). Almost all this calcium goes into eggshell formation, as egg yolk contains a negligible amount of calcium (Etches, 1987). The 2.2 g eggshell calcium mostly comes directly from the food source, but 20–40% comes from bone (Bar, 2009). Therefore, laying hens need to export about 0.44–0.88 g calcium from their skeleton in every laying cycle. Medullary bone is absorbed and renewed rapidly (Bain et al., 2016), and mobilizes calcium at a much higher rate than the cortical bones and femur ends (Hurwitz, 1965). Most bone-sourced eggshell calcium is therefore from medullary bone. Hurwitz (1965) fed laying hens using Ca^45^-labelled diet and found that calcium turnover rate of medullary bone is about 10 times higher than that of cortical bones. Moreover, he discovered that about 70% of calcium in the femur and tibia medullary segments was renewed within a 12 day period in laying hens. The high calcium turnover rate of medullary bone is associated with its structure. Compared with cortical bone, medullary bone has a lower mineral concentration, lower mineral crystallinity and thinner, shorter and less organized mineral particles (Kerschnitzki et al., 2014; Nys and Le Roy, 2018).

### Bone blood flow for growth and locomotion

Femoral nutrient foramen size in relation to femoral bone blood flow was previously correlated with bone growth in growing animals. Intraspecifically, growing animals may require a relatively higher blood flow rate to long bones to support primary bone modelling. Long bones of younger mammals have a higher relative blood flow rate than those in older ones, consistent with higher metabolic rates during growth (Nakano et al., 1986; Pasternak et al., 1966; Whiteside et al., 1977). Femoral nutrient foramen sizes of in-pouch kangaroo joeys are severalfold larger than in adult marsupial species of similar body mass (Hu et al., 2018). All chickens in our study were still growing to some extent, so they may have been influenced by elevated bone perfusion for growth. Younger non-laying hens tended to have relatively larger foramen sizes than older laying hens (Fig. 3), and the mass-independent values were significantly different (Fig. S2). Although the chickens in this study were selected to have similar ages around the onset of sexual maturity, in order to avoid bone growth differences among groups, the ages among the three chicken groups were still slightly different and they were all much younger than the chickens studied previously. Greater perfusion for bone growth is also implied by the higher wet bone mass-specific blood flow to the femur ends, compared with the shaft (Table 1), as the secondary ossification centres are located at the ends of long bones (Brookes and Revell, 1998).

Femora are responsible for absorbing stress from weight bearing and locomotion. The microfractures on bones caused by stress can be repaired by energy-driven Haversian remodelling (Lieberman et al., 2003). Femoral bone blood flow is therefore also related to animal locomotor activity levels in adult terrestrial vertebrates. Interspecifically, terrestrial vertebrates with higher maximum metabolic rates and higher activity levels tend to have relatively larger femoral nutrient foramen sizes (Allan et al., 2014; Seymour et al., 2012). The femoral bone perfusion rates of the non-laying hens and roosters were not significantly different. This probably suggests that they have no great difference in locomotion intensity, without considering the minor effects of calcium mobilization and growth rate differences between the two groups. The higher perfusion rates in chicken femur ends than the shaft may relate to higher oxygen demand in these regions. Femur ends include both metaphyses and epiphyses, which are supplied by a great number of arteries. Some foramina at the ends are larger than the shaft nutrient foramen in mammals (Brookes and Revell, 1998), suggesting long bone ends contain larger arteries and thus require higher blood flow rates. The femur ends may require more energy for undergoing the remodelling process than the shaft, as they are located near the joints and experience more intense stress during daily activity.

### Relating nutrient foramen size to femoral bone blood flow

**Fig. 5.**
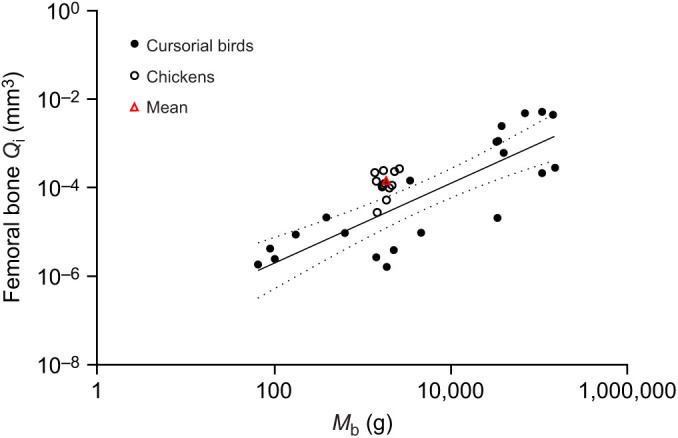
**Scaling of femoral bone blood flow index (**
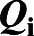
**) on *M*_b_ of cursorial birds including chickens.** Data are mean 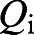 values of 21 cursorial bird species, and individual and mean 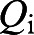 values of the 12 subadult chickens from this study. The equation of the regression is 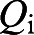
=3.2×10^−8^*M*_b_^0.90±0.29^ (*R*^2^=0.68, *P*=0.03), and it includes the single mean value for chickens (red triangle). The dotted lines represent the 95% CI for regression mean. Data for the 21 bird species were obtained from Allan et al. (2014).

Despite the microsphere experiment showing that laying hens have a higher blood flow rate of femoral bone and femur shaft than the non-laying hens, laying hens had relatively smaller absolute and mass-independent foramen sizes than the non-laying hens (Fig. 3; Fig. S2). No significant differences in foramen size occurred between non-laying hens and roosters. We expected that the higher femoral bone blood flow in laying hens would correlate with larger, rather than smaller foramen sizes. All previous studies on long bone nutrient foramina assumed that foramen size is proportional to the occupying artery size, which represents the amount of supplying blood flow. Previous foramen studies also assumed the size ratio between the foramen and artery remains constant interspecifically and intraspecifically. In this study, no correlation was found between nutrient foramen radius and femoral bone blood flow (Pearson's *r*=−0.11, *P*=0.68) (Fig. 4). In contrast, our companion study involved another group of chickens in which imaging of nutrient arteries filled under physiological pressure with a contrast medium (BriteVu) showed a significant positive correlation between foramen and artery lumen size (Hu et al., 2021). It should be noted that the 18 chicken samples in this study only cover a 2.5-fold body mass range. Coupled with high variability in the data, this range is too narrow to reveal any correlation between foramen size and blood flow rate, as allometric studies generally require body mass ranges of 100-fold or more. However, if we consider the results for chickens in this study to represent a single value for *Gallus domesticus*, we can compare it with an interspecific allometric analysis of over 1000-fold range of body mass in adult cursorial birds (Fig. 5). The mean 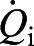 was 1.50×10^−4^ mm^3^, which is higher than the 95% confidence bands for adult birds in general. This probably relates to the fact that our chickens were actually still growing, because bone growth is associated with relatively larger nutrient foramina (Hu et al., 2018). At present, the best correlation between bone shaft blood flow rate (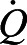, ml min^−1^) and nutrient foramen radius (*r*, mm) is 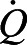/*r*=1.07 ml min^−1^ mm^−1^ (Fig. 4). This absolute value may replace the relative bone perfusion index, 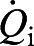, in future evaluations of femoral blood flow rate based on nutrient foramen size alone.

## Supplementary Material

Supplementary information
