# Supplementary material for: Regional femoral bone blood flow rates in laying and non-laying chickens estimated with fluorescent microspheres
Source: J Exp Biol. 2021 Aug 20;224(16):jeb242597. doi: 10.1242/jeb.242597 (PMC8407662; doi:10.1242/jeb.242597)
Supplement: Supplementary information [file jexbio-224-242597-s1.pdf]

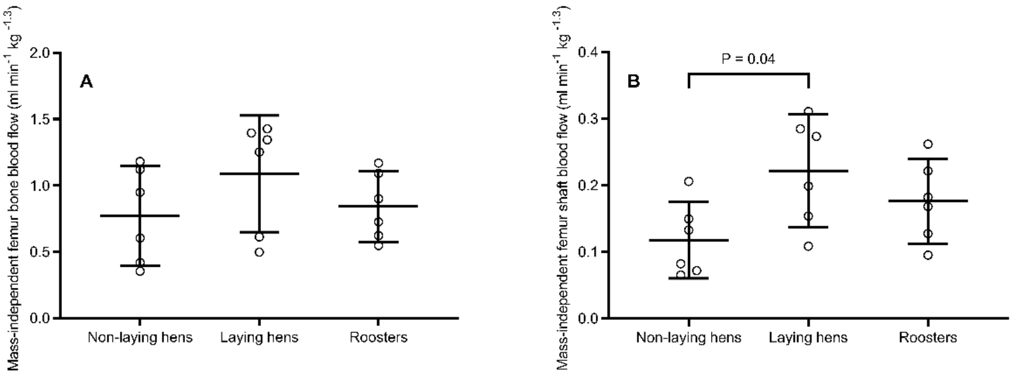

Fig. S1. Mass-independent blood flow rates of femoral bone (A) and femur shaft (B) among non-laying hens, laying hens and roosters. Mass is body mass to the 1.3 power. Error bars represent 95% confidence interval (CI) of the means of 6 replicates.

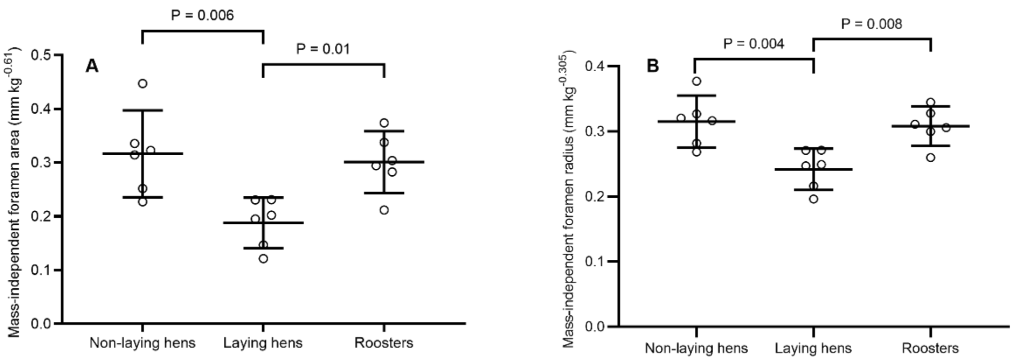

Fig. S2. Mass-independent nutrient foramen areas (A) and radii (B) of non-laying hens, laying hens and roosters. Error bars represent 95% confidence interval (CI) of the means of 6 replicates.

Journal of Experimental Biology • Supplementary information

Table S1. Average and 95% confidence intervals of mass-independent blood flows in the femoral bone and regional femoral bone of non-laying hens, laying hens, roosters and all 18 chickens. Mass-independent blood flow rate is calculated by dividing absolute blood flow values by the body mass to the 1.3 power. All data are averaged from both left and right femora.

	Mass-independent blood flow rate (ml min^{-1} $kg^{-1.3}$)
**Non-laying hens**	
Average femur	0.77 ± 0.38
Femur proximal end	0.28 ± 0.14
Femur shaft	0.12 ± 0.06
Femur distal end	0.38 ± 0.19
**Laying hens**	
Average femur	1.08 ± 0.44
Femur proximal end	0.33 ± 0.14
Femur shaft	0.22 ± 0.08
Femur distal end	0.53 ± 0.24
**Roosters**	
Average femur	0.84 ± 0.27
Femur proximal end	0.27 ± 0.12
Femur shaft	0.18 ± 0.06
Femur distal end	0.39 ± 0.11
**All 18 chickens**	
Average femur	0.90 ± 0.18
Femur proximal end	0.30 ± 0.06
Femur shaft	0.17 ± 0.04
Femur distal end	0.43 ± 0.09

Journal of Experimental Biology • Supplementary information

Supplementary Materials and Methods

Mass-independent results

Absolute blood flow of femoral bone scales with body mass with a scaling exponent of 1.3 ± 0.93. (Fig. 1). Wet bone mass-independent blood flow values were therefore calculated by dividing body mass to the 1.3 power. Wet bone mass-independent blood flow rates of femoral bone are not significantly different among three chicken groups ($F_{2, 15}$ = 1.3, P = 0.29), but laying hens have significantly higher wet bone mass-independent blood flow rates of shaft bone than the non-laying hens (P = 0.04, 95% confidence interval (CI) of the difference 0.10 ± 0.10 ml min^{-1} $kg^{-1.3}$) (Figure S1A & B).

Wet bone mass-independent blood flow rate in the femoral bone of laying hens is 1.08 ± 0.44 ml min^{-1} $g^{-1.3}$. The mean wet bone mass-independent blood flow rate in the femoral bone of laying hens is approximately 1.4 times higher than the non-laying hens, and the mean blood flow in the femur shaft is 1.8 times higher. Wet bone mass-independent blood flow rates of each femur section among three chicken groups were calculated and summarized in Table S1.

Femoral bone blood flow index ($Q_i = r^4/L$) in the femoral bone of cursorial birds scales with body mass to the 0.89 power (Allan et al., 2014). Any lengths of objects scale with their volumes (or masses) to the 0.33 power, if the shapes of the objects are the same. Assuming that Q_i is proportional to body mass to the 0.89 power, L to the 0.33 power, foramen radius should scale with body mass to the 0.305 power, and area to the (0.305 × 2 = 0.61). To compare foramen sizes among three groups, mass-independent foramen areas (mm^2 $kg^{-0.61}$) and radii (mm $kg^{-0.305}$) were calculated and compared using ANOVA.

Laying hens had significantly smaller mass-independent areas and radii of nutrient foramen than the non-laying hens (Area: P = 0.006, 95% CI of the difference -0.13 ± 0.09 mm^2; Radius: P = 0.004, 95% CI of the difference -0.07± 0.05 mm) and roosters (Area: P = 0.01, 95% CI of the difference -0.11 ± 0.09 mm^2; Radius: P = 0.008, 95% CI of the difference -0.07 ± 0.05 mm), while no significant differences in mass-independent foramen area or radius were observed between the non-laying hens and roosters (Fig. S2A & B).

Journal of Experimental Biology • Supplementary information
